# Response to sunitinib of a gastrointestinal stromal tumor with a rare exon 12 *PDGFRA* mutation

**DOI:** 10.1186/s13569-015-0036-9

**Published:** 2015-09-21

**Authors:** Andrew S. Brohl, Elizabeth G. Demicco, Karen Mourtzikos, Robert G. Maki

**Affiliations:** Sarcoma Department, Moffitt Cancer Center, 12902 Magnolia Drive, Tampa, FL 33612 USA; Department of Pathology, Icahn School of Medicine at Mount Sinai, New York, NY USA; Zwanger-Pesiri Radiology, Lindenhurst, NY USA; Hematology and Medical Oncology, Icahn School of Medicine at Mount Sinai, New York, NY USA

**Keywords:** GIST, PDGFRA, Imatinib, Sunitinib

## Abstract

**Background:**

Gastrointestinal stromal tumors (GISTs) are commonly driven by activating mutations in either *KIT* or *PDGFRA*. Importantly, different mutations within these two genes can lead to very different levels of sensitivity or resistance to kinase inhibitor therapy. Due to rarity, sensitivity or resistance of exon 12 *PDGFRA* mutant GIST to kinase inhibitor therapy is not well defined.

**Case summary:**

We report the case of a patient with a *PDGFRA* exon 12 mutated GIST. The patient experienced a very good response to imatinib in the neoadjuvant setting, but then relapsed while still on adjuvant imatinib. In this patient, we report a dramatic response to second line treatment with sunitinib, with complete resolution of two liver lesions at the time of first restaging.

**Conclusions:**

This is the first report detailing a response to treatment with sunitinib of a gastrointestinal stromal tumor with an uncommon exon 12 *PDGFRA* mutation. Based on the observed efficacy, GIST patients with this rare molecular subtype should be considered for sunitinib therapy.

## Background

Gastrointestinal stromal tumors (GISTs) are commonly driven by activating mutations in either *KIT* or *PDGFRA* [1, 2]. These molecular targets have successfully been exploited by treatment with small molecule kinase inhibitor therapy to dramatically impact clinical outcomes [3]. Importantly, different mutations within these two genes can lead to very different levels of sensitivity or resistance to inhibitor therapy [4]. It is therefore essential for researchers and clinicians to keep an accurate catalog of how different mutations respond to therapeutics.

Occurring in only 5–7 % of GIST, *PDGFRA* mutations are by far the less common of the two driving kinase gene mutations in GIST. *PDGFRA* mutations are found predominantly in exons 18 (~5 %), 12 (~1 %) and 14 (<1 %), and over half of all *PDGFRA* mutations involve the same amino acid change in exon 18, D842V [2]. The *PDGFRA* D842V mutation confers imatinib resistance in vitro as well as in clinical observations [1, 2, 5]. Due to rarity, other *PDGFRA* mutations have not been well characterized in individual treatment responsiveness, but collectively are considered sensitive to imatinib therapy based on in vitro data, clinical reports and improvement in outcomes in the “non-D842V *PDGFRA* mutation” catch-all subset that is often used in reporting [2, 6]. For the rare *PDGFRA* exon 12 mutations, the largest retrospective series to date reported a 50 % objective response rate to imatinib in 8 GIST cases with an exon 12 mutation [5]. Additionally, 1 of 1 patient with *PDGFRA* exon 12 mutation on a phase III clinical trial, and 1 additional case report of GIST with exon 12 mutation noted a good response to imatinib [7, 8].

Sunitinib is commonly used as second line therapy for GIST after imatinib failure. Very few *PDGFRA* mutant GIST patients with sunitinib treatment outcomes have been reported to date and to our knowledge no significant objective responses have been reported in this subset of GIST patients [5, 9–11]. The majority of *PDGFRA* mutations reported in these clinical series, however, have been of the D842V variety, which based on in vitro data would not be expected to have substantial activity to sunitinib treatment [11].

We report the case of a patient with *PDGFRA* exon 12 mutated GIST. The mutation in our patient’s tumor results in an in-frame deletion of amino acids 559 and 560, p.(W559-R560del). This deletion has not previously been reported, though is adjacent to a hotspot for mutation in exon 12, V561D, and is partially overlapping with previously reported deletions [2, 12]. We report the clinical responses to neoadjuvant imatinib as well as to second line therapy for relapsed disease with sunitinib. To our knowledge this is the first case reporting on treatment response of *PDGFRA* exon 12 mutated GIST in these two scenarios.

## Case presentation

A 48-year-old male without significant past history was evaluated in December 2010 for abdominal pain. An abdominal sonogram showed a large heterogeneous mass originating from the stomach. PET/CT scan confirmed a 21 × 13 × 9 cm gastric mass with moderate ^18^F-fluorodeoxyglucose (FDG) uptake, SUV_max_ 6.0. Endoscopic ultrasound with biopsy was performed January 2011 and pathology was consistent with a gastrointestinal stromal tumor (GIST). The patient was treated with neoadjuvant imatinib 400 mg daily. With treatment, the mass significantly reduced in size to 9.2 × 6.8 × 4.9 cm on pre-operative imaging in September 2011. Laparoscopic resection was performed November 2011, with pathology confirming an 8.5 cm gastric GIST with focal myxoid changes and extensive post-treatment changes. No mitoses were identified in this post treatment specimen. Immunohistochemistry was positive for CD117 and negative for CD34, S-100, SMA, desmin, and cytokeratin. Genetic testing was performed on the tumor specimen and it was found to lack *KIT* mutation by direct sequencing of exons 9, 11, 13 and 17.

Our patient continued on treatment with adjuvant imatinib 400 mg daily despite having bothersome but low grade toxicity including fatigue, diarrhea, abdominal cramping, edema and joint pains. While still on adjuvant imatinib, surveillance imaging in September 2013 revealed a new 1.2 cm nodule in the mesentery. Follow-up PET imaging showed mild FDG uptake, SUV 1.9. Resection was performed November 2013, and pathology was consistent with recurrent GIST measuring 1.7 cm. The post-surgical course was complicated by a symptomatic excisional hernia. At this recurrence, the resection specimen was sent for repeated gene mutation screening, this time for both *KIT* and *PDGFRA*, and an exon 12 deletion, p.(W559-R560del), was detected in *PDGFRA*. No mutation was found in *KIT*. Resumption of kinase inhibitor therapy either at dose escalation [13] or switch to an alternate agent [14] was considered post-operatively but was declined by the patient due to quality of life concerns.

**Fig. 1:**
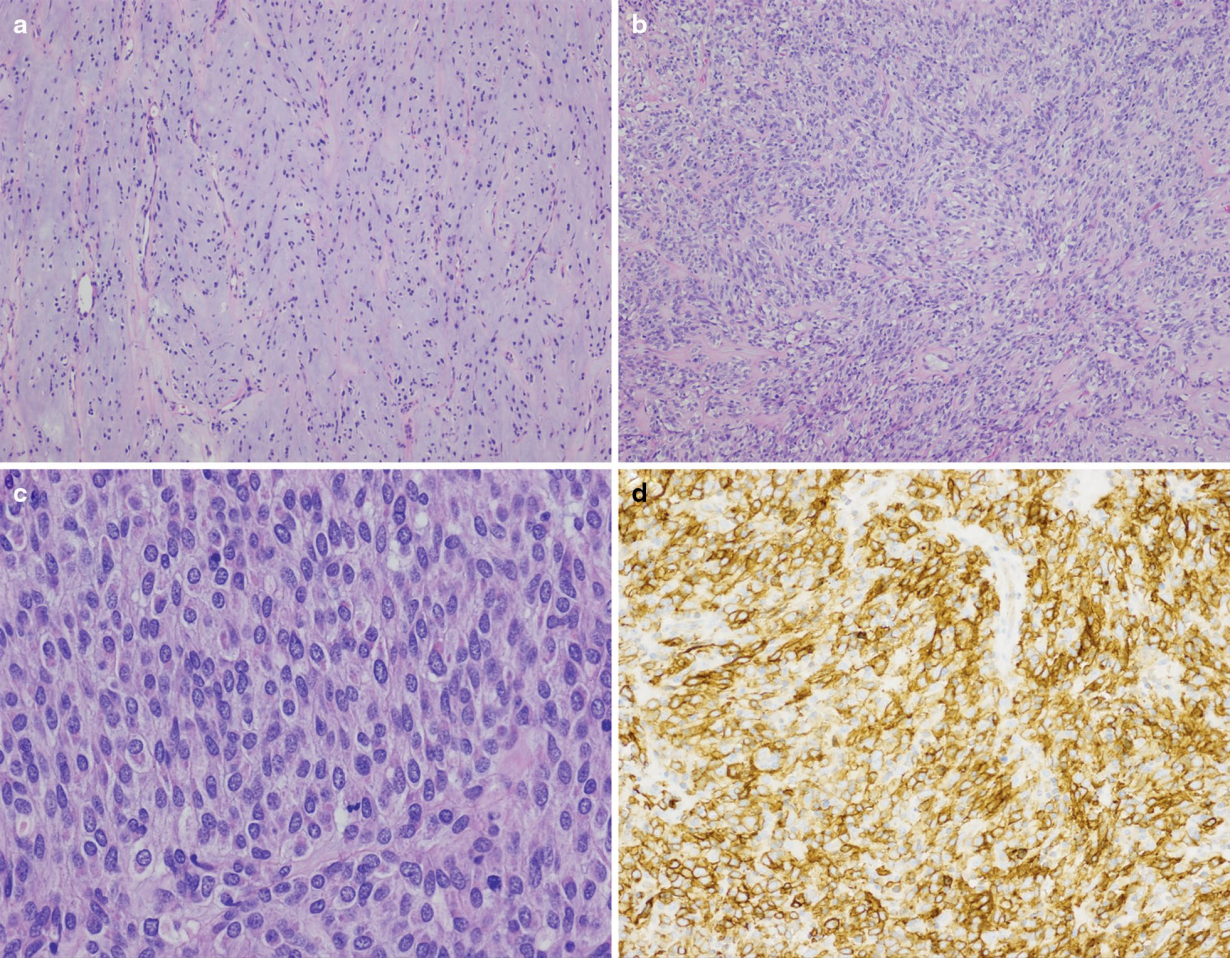
At low power (**a**, **b**, ×100), the metastatic GIST resected in 2014 demonstrated variable cellularity, with spindled to predominately epithelioid cells embedded within a myxoid stroma. On high power (**c**, ×400) cellular areas demonstrated sheets of epithelioid tumor cells with abundant cytoplasm and monotonous nuclei with finely granular chromatin, typical for epithelioid GIST. Scattered mitotic figures were present. Immunohistochemical study for DOG1 (**d**, ×200) was diffusely positive in tumor cells

In June 2014, CT imaging revealed a new 1.9 × 1.7 cm abdominal nodule abutting the undersurface on the left diaphragm, consistent with second recurrence. Laparoscopic resection with excisional hernia repair was undertaken September 2014. During this procedure, the patient was noted to have two additional small tumor implants in the anterior abdominal wall. All three lesions were excised and consistent with metastatic GIST (Fig. 1). At post-operative restaging imaging done December 2014, the patient was noted to already have a new 4.2 cm liver lesion and a smaller 5 mm liver lesion. Shortly thereafter, he developed severe RUQ abdominal pain and was hospitalized for hemorrhage of the liver lesion and concurrent drop in hemoglobin requiring transfusion, but was managed conservatively with resolution over several days.

**Fig. 2:**
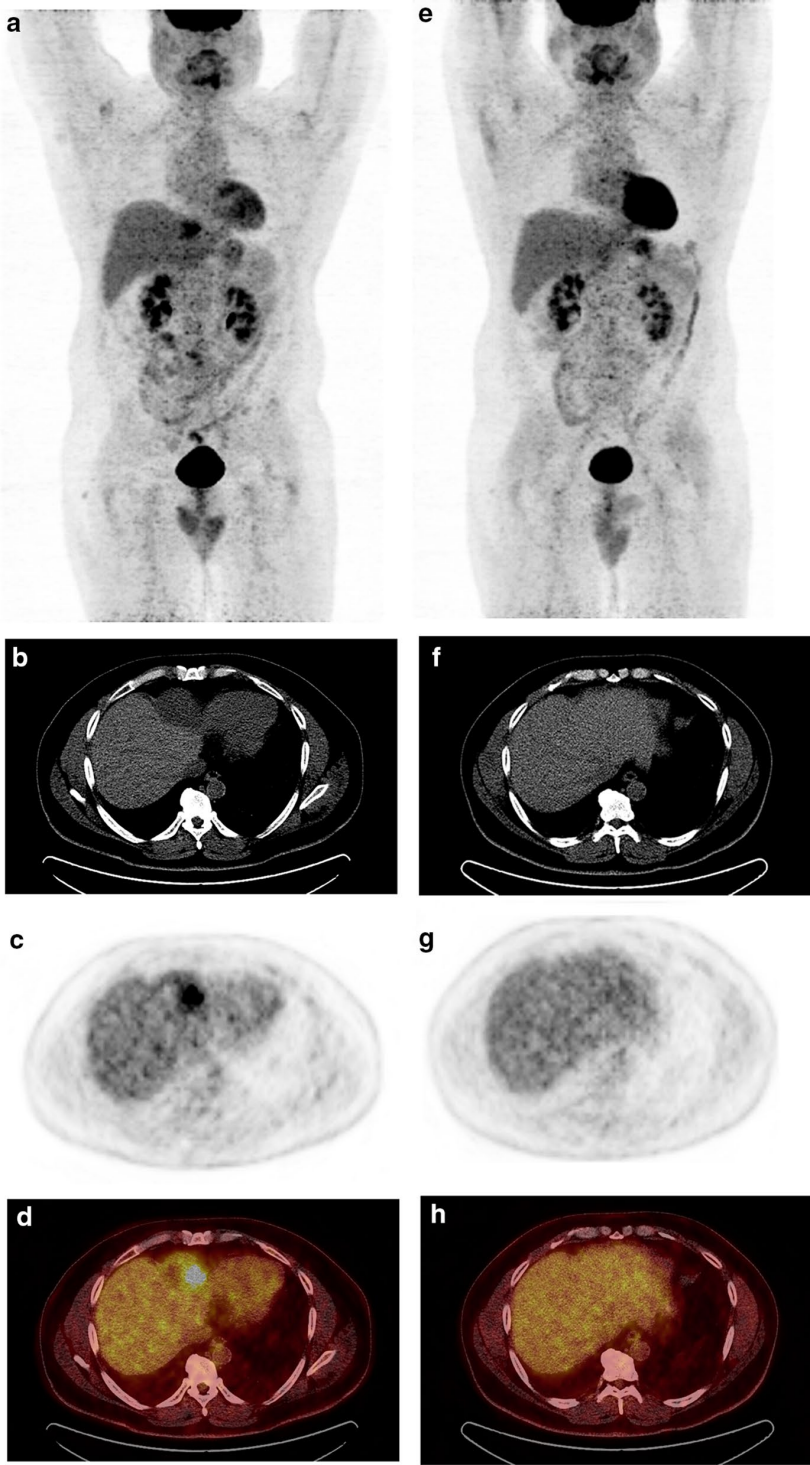
PET/CT imaging prior to (**a**–**d**) and after 10 weeks (**e**–**h**) of sunitinib therapy. Prior to treatment, whole body PET (**a**) revealed a large hypermetabolic lesion in the dome of the liver, a PET-avid sub-cm L hepatic lesion, and hypermetabolic activity in the abdominal wall near the site of previous surgical resection. On cross sectional CT imaging (**b**) the large hepatic lesion measured 5.9 × 5.1 cm in maximal dimension, and the corresponding area on PET imaging (**c**) and fused PET/CT (**d**) revealed an SUV_max_ of 8.4. Both liver lesions were no longer detectable on repeat imaging (**e**–**h**), and the surgical site hypermetabolic activity decreased (**e**), consistent with resolving post-operative changes

The patient was started on systemic therapy for metastatic disease with sunitinib 25 mg daily in January 2015. A lower than typical starting dose of sunitinib was selected due to concern for potential toxicity in light of recent hemorrhage [14]. Prior to the start of therapy, he was restaged with PET/CT, showing FDG avid lesions in the liver dome (5.9 × 5.1 cm, SUV 8.4) and a small left liver focus (sub-cm, SUV_max_ 5.2). There additionally was PET-avidity (SUV_max_ 5.9) in the anterior abdominal wall at the site of recent surgical resection and repair. On first restaging imaging after 10 weeks of treatment, PET/CT imaging revealed a dramatic improvement with the large hypermetabolic liver lesion no longer visible, nor was visible the smaller hepatic focus of FDG uptake (Fig. 2). The area of hypermetabolic activity in the anterior abdominal wall decreased in size and metabolic activity, radiographically favoring partial resolution of postoperative change though cannot rule out partial regression of metastatic disease. Sunitinib therapy is ongoing and has been well tolerated with grade 1 hypertension, grade 1 skin pallor and grade 1–2 generalized fatigue noted as potential toxicities.

## Conclusions

We report the case of a gastrointestinal stromal tumor with a previously unreported exon 12 deletion in *PDGFRA*. The patient was treated with neoadjuvant imatinib with reduction in size of the initial tumor from 21 cm in greatest dimension at the start of therapy to 8.5 cm at the time of resection. Responsiveness to imatinib is consistent with a previous series that reported a 50 % response rate to first line imatinib in a subset of 8 GIST patients with exon 12 *PDGFRA* mutation [5]. Unfortunately, despite a very good response to imatinib in the neoadjuvant setting, our patient relapsed while still on continued adjuvant imatinib therapy. The approximate 2 year time between imatinib sensitivity and development of recurrence in our patient is similar to the typical time frame reported for the development of secondary imatinib resistance in the metastatic setting [11].

Most notably, we observed a striking response to treatment with sunitinib in this patient with an exon 12 *PDGFRA* mutation, with complete resolution of two liver lesions at the time of first restaging. To our knowledge, this is the first reported objective response to sunitinib in *PDGFRA* mutated GIST in the literature. This favorable response is in conflict with the few previous reports on this subject, which have typically reported very limited efficacy of sunitinib in patients with *PDGFRA* mutated GIST [5, 9–11, 15]. The majority of *PDGFRA* mutations reported in these clinical series, however, have been of the D842V variety, which based on in vitro data would not be predicted to have substantial activity to sunitinib treatment [11].

A limitation of our current report is that molecular testing in this case was restricted to that used for clinical practice and *PDGFRA* mutation testing was not implemented on every specimen. At the time of initial resection, only *KIT* mutational testing was performed. It is therefore not definitive that the *PDGFRA* exon 12 deletion was present at the time of initial imatinib response. *PDGFRA* exon 12 mutation, however, is most consistent with an imatinib-sensitive primary driver mutation [1, 2, 5], rather than a secondary resistance mutation, which arise in the ATP binding pocket or loop domain of *KIT* or *PDGFRA* in response to the selective pressure of imatinib therapy [4, 15]. It is therefore likely that the *PDGFRA* mutation in our patient represents a primary driver mutation that was present throughout the disease course.

In summary, we report a dramatic response to sunitinib of a gastrointestinal stromal tumor with a rare exon 12 *PDGFRA* mutation. Compared to the previous literature, our case highlights the substantial difference in clinical activity of this agent in different mutational subtypes of GIST, even within the narrower category of *PDGFRA* mutant GIST. Our report adds to the growing catalog of mutation-to-drug efficacy links and fills a knowledge gap in one of the rare molecular subtypes. Though confirmatory reports are needed, sunitinib should be considered for the rare patient with *PDGFRA* exon 12 mutated GIST.

## Consent

Written informed consent was obtained from the patient for publication of this Case Report and any accompanying images. A copy of the written consent is available for review by the Editor-in-Chief of this journal.
